# Microsphere-assisted, nanospot, non-destructive metrology for semiconductor devices

**DOI:** 10.1038/s41377-022-00720-z

**Published:** 2022-02-07

**Authors:** Soonyang Kwon, Jangryul Park, Kwangrak Kim, Yunje Cho, Myungjun Lee

**Affiliations:** Equipment R&D Team 4, Mechatronics Research, Samsung Electronics Co., Ltd., 1-1 Samsungjeonja-ro, Hwaseong-si, Gyeonggi-do 18848 Republic of Korea

**Keywords:** Optical spectroscopy, Optics and photonics

## Abstract

As smaller structures are being increasingly adopted in the semiconductor industry, the performance of memory and logic devices is being continuously improved with innovative 3D integration schemes as well as shrinking and stacking strategies. Owing to the increasing complexity of the design architectures, optical metrology techniques including spectroscopic ellipsometry (SE) and reflectometry have been widely used for efficient process development and yield ramp-up due to the capability of 3D structure measurements. However, there has been an increasing demand for a significant reduction in the physical spot diameter used in the SE technique; the spot diameter should be at least 10 times smaller than the cell dimension (~30 × 40 μm^2^) of typical dynamic random-access memory to be able to measure in-cell critical dimension (CD) variations. To this end, this study demonstrates a novel spectrum measurement system that utilizes the microsphere-assisted super-resolution effect, achieving extremely small spot spectral metrology by reducing the spot diameter to ~210 nm, while maintaining a sufficiently high signal-to-noise ratio. In addition, a geometric model is introduced for the microsphere-based spectral metrology system that can calculate the virtual image plane magnification and depth of focus, providing the optimal distance between the objective lens, microsphere, and sample to achieve the best possible imaging quality. The proof of concept was fully verified through both simulations and experiments for various samples. Thus, owing to its ultra-small spot metrology capability, this technique has great potential for solving the current metrology challenge of monitoring in-cell CD variations in advanced logic and memory devices.

## Introduction

The rapid and precise imaging of three-dimensional (3D) semiconductor devices is of significant importance for semiconductor wafer inspection during the manufacturing process. In general, imaging techniques can be classified into two types, namely two-dimensional (2D) inspection and 3D inspection^1,2^. Scanning electron microscopy (SEM) and transmission electron microscopy (TEM) are techniques that can identify defects and measure the critical dimension (CD) of fine patterns in high-resolution 2D images^3,4^. In volumetric 3D imaging, spectroscopic analysis techniques, such as spectroscopic reflectometry (SR) and spectroscopic ellipsometry (SE), are widely used to detect 3D structural defects and measure various CDs in semiconductor devices simultaneously, owing to their advantages of high measurement speed, low cost, and minimal sample damage^5–8^. In addition, several notable spectroscopic methods for 3D metrology have been proposed to further improve the metrology sensitivity and precision through extension to the Mueller matrix and interferometric analysis, as well as the utilization of the wider wavelength ranges, including infrared (IR), extreme ultraviolet (EUV), and X-rays^9–15^. In all these inspection methods, identifying the defects in the early stages of the semiconductor manufacturing process is crucial to optimize the fab process control while reducing the operation cost.

Despite the importance of high-resolution 2D imaging-based inspection in manufacturing processing, the field of view (FOV) of SEM and TEM limits the inspection speed and throughput. Throughput refers to the productivity of equipment in the semiconductor manufacturing process and is generally defined as the number of processed wafers per hour. These techniques require a greater inspection time than that of spectral measurement systems to measure the entire area of an inspection wafer, and consequently, yield a lower throughput. In addition, the measurement of vertical dimensions in 3D device structures requires a destructive sample preparation process, which damages expensive wafers^16^. Unlike the SEM and TEM techniques, the spectroscopic inspection technique is a non-destructive metrology method, which can provide 3D structure information based on the spot measurement without damaging the wafers. However, the size of the illumination spot must be smaller than the size of the target device, where the typical spot diameter varies from 30 to 50 μm depending on the spectral range of measurements. Recently, smaller nanospot measurements have become highly desirable to measure CDs from both the edge and corner areas in unit cell blocks of dynamic random-access memory (DRAM) and static random-access memory (SRAM) devices. However, it is difficult to measure small corner areas in memory unit cells with a dimension of 20–40 μm in the *x*- and *y*-directions using conventional SE and SR systems owing to the relatively large illumination spot. Furthermore, reducing the measurement area significantly complicates the optical system^17–21^.

Various imaging approaches to break the optical resolution limit have been developed, such as fluorescence microscopy, stochastic optical reconstruction microscopy, stimulated emission depletion microscopy, and other techniques^22–24^. Although they are widely used for biomedical applications, they are not appropriate for semiconductors owing to the restrictions on fluorescent materials, destructive methods, and transmission microscopy. In recent years, the emerging microsphere-assisted nanoscopy has demonstrated the possibility of observing nanostructures beyond the Rayleigh limit^25–32^. A microsphere has a spherical shape with a radius of 1–50 µm, typically made of transparent and dielectric materials^33–36^. There are several theoretical models that describe how microspheres magnify objects and overcome the optical limit using white-light sources. One of the most interesting models that describe resolution enhancement based on microspheres is the photonic nanojet effect^37–42^. A photonic nanojet is an electromagnetic beam that is generated on the far side of the microsphere, which is known for being able to convert evanescent waves into propagating waves. This is one of the major theories regarding super-resolution in microspheres. Although the exact mechanism of super-resolution remains unknown, microsphere-assisted super-resolution techniques can be practically applied in various optical measurement systems, such as interferometry and confocal microscopy^43–48^.

In this paper, we present a microsphere-assisted spectroscopic reflectometry (MASR) system based on the combination of super-resolution imaging and SR methods. The MASR system not only surpasses the optical resolution limit for normal white-light conditions but also achieves spectral measurements with a nanospot diameter of 210 nm, while simultaneously maintaining an acceptable signal-to-noise ratio (SNR). The MASR system has a six times higher spectral intensity than the conventional ×100 SR, resulting in an improved SNR.

In addition to the MASR system, a geometric model of the virtual imaging field is presented to quantitatively define the optimal position of the microsphere between the objective lens and sample to be imaged. The image contrast and magnification of super-resolution images based on the microsphere varies with the distance from the microsphere to the objective lens or the sample. Previous studies have focused on understanding the super-resolution mechanisms induced by the photonic nanojet effect, while quantitative analysis is lacking. In other words, there is insufficient research data on the relationship between the microsphere position and corresponding image qualities of magnified images, including image contrast and magnification^49^. In this study, a novel geometric relationship is introduced to calculate the optimal positions of microspheres with various materials and diameters. Consequently, the best image quality can be obtained with the desired super-resolution magnification and FOV, maintaining a non-contact measurement condition for semiconductor devices.

The MASR system has the potential to solve current metrology and inspection challenges in advanced semiconductor systems. To the best of our knowledge, this study is the first to demonstrate the combination of super-resolution and spectral reflectometry techniques for semiconductor device measurement. This method can be applied to monitor in-cell structure variations in extremely small areas. In this study, the usefulness of the MASR system was experimentally validated by obtaining spectra and super-resolution images of semiconductor devices, which cannot be measured and detected using conventional spectral measurement systems and microscopes owing to the restricted area or nanostructures.

The remainder of this paper is organized as follows. The second section describes the experimental set-up of the MASR system used in this study. In the third section, the geometric model for super-resolution imaging and the nanospot spectral measurement are introduced and experimentally validated. The results are discussed in the fourth section. Finally, the fifth section presents the materials and methods used in this study.

## Experimental set-up of MASR

**Fig. 1:**
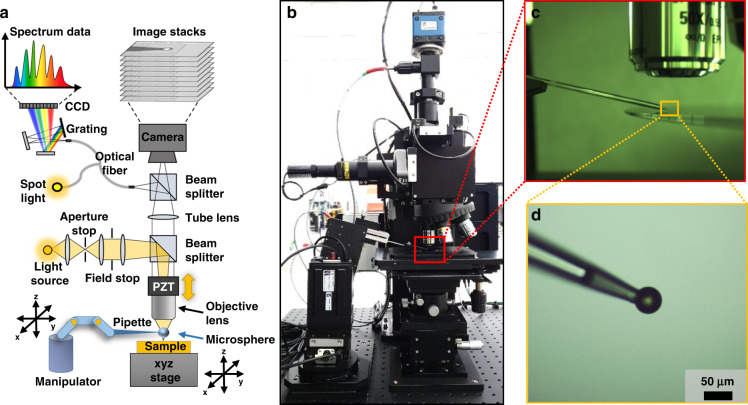
Configuration of the MASR system. **a** Schematic of the MASR system based on a conventional microscopy configuration. A white-light light-emitting diode (LED) was used as the light source, and aperture and field stops were used. The light illuminated the objective lens, microsphere, and sample sequentially. The reflected light from the sample was split between the image sensor to capture the image and the grating to analyze the spectrum data. **b** Imaging optics with the microsphere under the objective lens. **c** The microsphere attached to the micropipette was controlled by the micromanipulator. **d** Enlarged image of the microsphere attached to the micropipette.

Figure 1 shows a diagram and photograph of the MASR system based on conventional microscopy. By adding a spectrometer in the imaging optics, an ultra-small spot reflectometry system could be built based on the super-resolution effect arising from the microsphere. In this architecture, a white-light light-emitting diode (LED) with a wavelength range of 430–700 nm was used as the light source and three lenses with two irises were used for the illumination beam shaping, where the two irises were used as aperture and field stops. The power of the light source was 5 W and the intensity was 4 mW/cm^2^ at the back aperture plane of the objective lens. The broadband light passing through the beam splitter illuminated the objective lens and microsphere sequentially and was then directed toward the sample to be imaged. Here, the microsphere could further magnify the sample image beyond the Rayleigh limit, where two types of soda lime glass (SLG) or polystyrene (PS) microspheres were evaluated in this study. It is important to note that the microsphere must be located in the optimal position between the sample and the objective lens to obtain a high-quality super-resolution image. Accordingly, the position of the objective lens was precisely controlled using a piezoelectric tube controller, and the position of the microsphere was controlled using a micromanipulator, as depicted in Fig. 1a. The objective lens turret was set up with the PZT scanner to carry a maximum of five objective lenses, and three objective lenses were prepared (namely, an Olympus LMPLFLN ×20, Nikon CF Plan EPI ELWD ×50, and Nikon CF Plan EPI ELWD ×100) for imaging different types of semiconductor devices with different magnifications. The microsphere was attached to the end of a micropipette so that its position could be precisely controlled by the manipulator, as illustrated in Fig. 1c. Although micropipettes are more commonly used for volumetric measurements, previous research has shown that they can also be used to manipulate microspheres^50^. Since a micropipette has a sharper tip than a glass rod (which is another method for manipulating microspheres), it can maneuver more freely between the objective lens and sample to be imaged. Thus, the microsphere attached to the micropipette was able to approach the sample without any interference with the objective lens, sample, or sample stage^50^. The micropipette had a sharp tip with a length of 4 mm and diameter of 10 μm. The total length of the micropipette was 50 mm. The broadband incident light illuminated samples producing multiple internal reflections in the thin film layers below the nanostructures of the top layer. The multiple reflections interfered with each other within the optical system including the microsphere and objective lens. After traveling to the first beam splitter, the broadband light passed through a tube lens, which had a ×1 magnification and an effective focal length of 220 mm, and was split into two beams. One beam was directed toward a scientific complementary metal-oxide-semiconductor image sensor with 6.5 × 6.5 µm pixels to capture the super-resolution image, whereas the other was directed to the optical fiber located in the imaging plane. The optical fiber with a 100-μm diameter core was coupled to the grating, which dispersed light spatially by wavelength, and the dispersed light was directed into the charged coupled device sensor. The sensor had 1044 pixel arrays with a wavelength range of 200–1100 nm (QE65 Pro, Ocean Optics, USA). MASR could provide the broadband spectra reflected from the spot with a diameter of 210 nm owing to the extremely high magnification (approximately ×530) enhanced by the microsphere.

## Results

### Geometric model of microsphere-assisted super-resolution

In this section, a novel framework that facilitates the application of the geometric model to the analysis of the microsphere-assisted super-resolution imaging system is presented. The microsphere projects a magnified virtual image into the far-field, and the image is collected by the objective lens. The working distance of the objective lens and the virtual image varies according to the materials and diameters of the microspheres. Additionally, the image contrast and magnification of the virtual image changes as a function of the working distance.

Several methods to improve the optical resolution using the microsphere and super-resolution principles have recently been verified experimentally^25–32^, while some have attempted to combine the microsphere with various optical measurement systems^43–48^. Furthermore, most research on imaging has been conducted for the case of direct contact between the sample and microsphere^30,31^. The super-resolution effect of the microsphere has been widely researched^33,38–40^; however, the geometric relationship between the sample and its magnified virtual image generated by the microsphere has not been fully explained and defined thus far^49^.

In this study, a geometric model was developed to determine the optimal distance from the microsphere to the sample and objective lens to maximize the super-resolution imaging performance. This model can aid in quantitatively determining the diameter and material of the microsphere for the desired magnification and defining the optimal non-contact position of the microsphere where the highest-contrast image can be acquired because it is vital to maintain the non-contact condition between the sample and microsphere for semiconductor metrology.

#### Virtual imaging plane and magnification rule

In this sub-section, the new framework is introduced to understand and analyze the photonic nanojet effect that arises from a microsphere for optimizing the MASR system. To observe the super-resolution image enhancement produced by the microsphere, a photonic nanojet, which is a narrow, high-intensity, and sub-diffraction waist beam, must be generated. The photonic nanojet propagates into the background medium from the far side of the microsphere, and can be considered as the focused energy point of the incident light^37–42^. However, this does not indicate that the objective lens needs to be focused on the position of the photonic nanojet^39,40^. Instead, the focus of the objective lens must be set in the virtual image plane, resulting in the observation of the super-resolution image in the microscopy system. This is because the actual focal length of the microsphere and the concentration feature of the light wave after passing through the microsphere behave significantly differently from those in the conventional system.

**Fig. 2:**
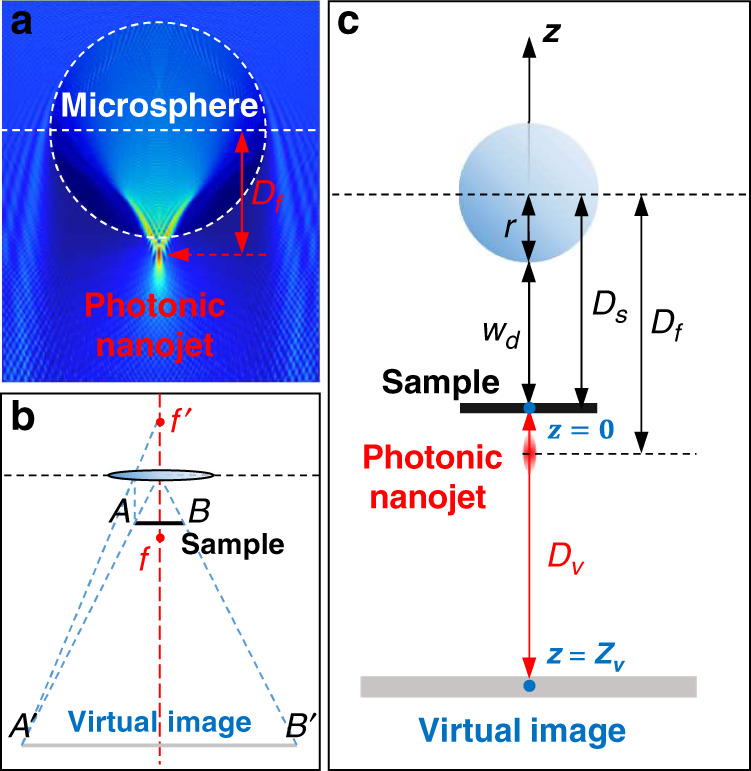
Formation of the virtual image and relationship between the photonic nanojet and the projected virtual image plane. **a** Simulated photonic nanojet effect (indicated by the red arrow) by finite-difference time-domain (FDTD) method. **b** Diagram combining ray optics and FDTD simulation. ($\overline{AB}$: sample. $\overline{A\text{'}B\text{'}}$: virtual image. $f$: photonic nanojet as a front focal point from FDTD. $f\text{'}$: back focal point. $T$: microsphere regarded as a thin lens.). **c** Geometric relationship between the microsphere, sample, and virtual image. ${D}_{{\rm{v}}}$: distance between the virtual image plane and sample. $({D}_{{\rm{v}}} > 0)$ ${D}_{{\rm{s}}}$: distance between the center of the microsphere and sample. $({D}_{{\rm{s}}} > 0)$ ${D}_{{\rm{f}}}$: distance between the microsphere and photonic nanojet, obtained by FDTD simulation. $({D}_{{\rm{f}}} > 0)$ ${w}_{{\rm{d}}}$: distance between the bottom of the microsphere and sample. *r*: radius of the microsphere. (${w}_{{\rm{d}}}\ge 0$). ${Z}_{{\rm{v}}}$: axial position of virtual image. $({Z}_{{\rm{v}}}=0-{D}_{{\rm{v}}})$.

Figure 2 depicts the imaging plane analysis according to the positions of the sample, microsphere, and virtual image to calculate the magnification and distance from the sample to the virtual image plane by classical ray optics using finite-difference time-domain (FDTD) simulation. Because the photonic nanojet effect shown in Fig. 2a cannot be fully explained by ray optics, the position of the photonic nanojet was calculated via FDTD simulation^37–42^. In the ray optics approach, the microsphere behaves as a thin lens with a single principal plane at its center. Therefore, the back and front focal lengths of the microsphere are defined as *f* and *f’*, respectively, based on the thin lens approximation of the microsphere, as shown in Fig. 2b.

The magnification, *M*, between $\overline{AB}$ and $\overline{A\text{'}B\text{'}}$ is defined according to the geometric relationship depicted in Fig. 2c. This magnification rule for the super-resolution of the microsphere can be expressed as follows:

1 $$M=\frac{\overline{{A}^{\text{'}}{B}^{\text{'}}}}{\overline{AB}}=\frac{{D}_{{\rm{s}}}+{D}_{{\rm{v}}}}{{D}_{{\rm{s}}}}=\frac{{D}_{{\rm{f}}}+{D}_{{\rm{s}}}+{D}_{{\rm{v}}}}{{D}_{{\rm{f}}}}$$

The distance between the microsphere and sample is the sum of *r* and *w*_d_. From the FDTD simulation, *D*_f_ is determined, and *D*_v_ can be derived using Eq. (1) as follows:

2 $${D}_{{\rm{v}}}=\frac{{D}_{{\rm{s}}}^{2}}{{D}_{{\rm{f}}}-{D}_{{\rm{s}}}}=\,\frac{{(r+{w}_{{\rm{d}}})}^{2}}{{D}_{{\rm{f}}}-(r+{w}_{{\rm{d}}})}$$

where the sample and microspheres can either be in contact (${w}_{{\rm{d}}}=0$) or not in contact (${w}_{{\rm{d}}}\, > \,0$).

**Fig. 3:**
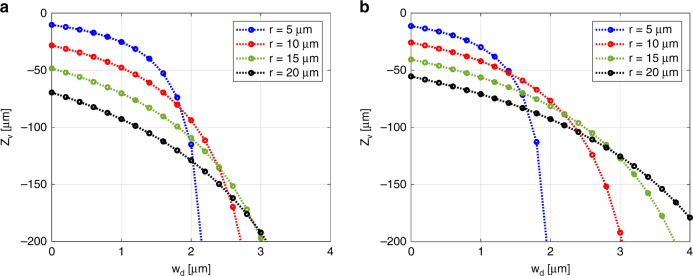
Simulated results of axial position *Z*_v_ vs *w*_d_ for microspheres with radii of 5, 10, 15, and 20 µm. **a** SLG (*n* = 1.52 and *λ* = 524 nm) and **b** PS (*n* = 1.60 and *λ* = 524 nm). *n* represents the refractive index for a specific wavelength *λ*. ${Z}_{{\rm{v}}}$ represents the relative position of the virtual image from the sample. As ${w}_{{\rm{d}}}$ increases, the magnified super-resolution image is formed at a lower position.

Figure 3a, b shows the variations of Z_v_ with ${w}_{{\rm{d}}}$ and *r*, calculated by Eq. (2), for various microspheres. It is feasible to calculate the distance between the virtual image and sample $\,({Z}_{{\rm{v}}})$ for the various microspheres, particularly in the cases where the sample and microsphere are not in contact $({w}_{{\rm{d}}}\, > \,0)$. For non-destructive inspection, it is important to calculate ${D}_{{\rm{v}}}$ and ${Z}_{{\rm{v}}}$ when the microsphere is not in contact with the target sample $({w}_{{\rm{d}}}\, > \,0)$ in Eq. (2). Therefore, rapid and precise positioning of the microsphere and objective lens can be performed to obtain the best possible quality image with desired magnification.

The magnification, *M*, is calculated from Eq. (1). Therefore, not only the theoretical magnification during imaging, but also the diameter of the spectral measurement area used in the MASR system can be calculated. Furthermore, it is possible to control the magnification by varying the vertical position of the microsphere or diameter or material of the microsphere.

#### Experimental results

To experimentally verify $M$ and optimal ${Z}_{{\rm{v}}}$ according to the proposed geometric model in Eqs. (1) and (2), it was necessary to measure the distance from the sample to the virtual image, ${D}_{{{\rm{v}}}_{\exp }}$, and the measured magnification, ${M}_{\exp }$. ${Z}_{{{\rm{v}}}_{\exp }}$ was identified by the axial position of the virtual image where the image contrast was highest in the vertical (*z*) direction. The magnification in the *x*–*y* plane measured from the image at ${Z}_{{{\rm{v}}}_{\exp }}$ was defined as ${M}_{\exp }$.

To measure ${Z}_{{{\rm{v}}}_{\exp }}$ and ${M}_{\exp }$, a 3D image stack was obtained by vertically scanning the objective lens while maintaining the microsphere in the same position. The image stack was made by capturing images, while changing the objective focal plane from the sample surface to 80 μm below the surface providing a sufficiently wide range to identify the position of the maximum image contrast. A scan interval of 0.08 μm was used, which provided a sufficient vertical resolution to determine the optimal position of the microsphere. ${Z}_{{{\rm{v}}}_{\exp }}$ was determined through image processing by calculating the highest edge sharpness of all the images in the stack using the Sobel filter. The total magnification of the super-resolution image acquired by the MASR system was the product of the enhanced magnification by the microsphere and optical system magnification determined by the objective and tube lenses. Therefore, the total magnification has to be divided by the optical system magnification to evaluate the microsphere magnification ${M}_{\exp }$. More details regarding image processing, image stack, and magnification are presented in the section “Vertical scanning and calculation of sharpness score and magnification”.

**Fig. 4:**
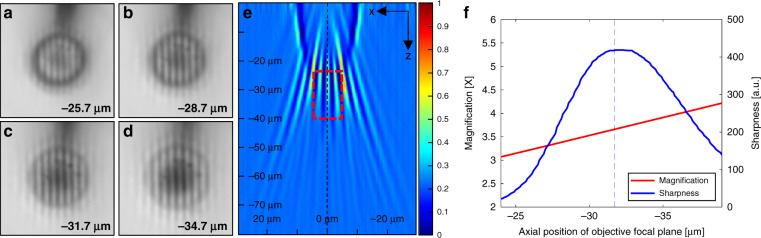
Image stack of standard grating patterns, consisting of a 0.35-μm line width and 0.7-μm pitch, gathered by the MASR system with a ×20 objective lens to experimentally verify the axial position of the virtual image *Z*_v_ and the magnification *M*. SLG microsphere with a radius of 12 μm was used. Images at different vertical positions (negative sign indicates a lower position from the sample): *z*: **a** −25.7 μm, **b** −28.7 μm, **c** −31.7 μm, and **d** −34.7 μm are shown. **e** *x*–*z* projection image of the image stack with the background signal removed. For analyzing the optimal position of the virtual image with the highest contrast, the edge sharpness in a specific region (red-dashed box) was calculated. **f** Variation in the image contrast (sharpness) and magnification according to the axial position of the objective focal plane. The experimental magnification was calculated by counting the number of pixels with the image processing software, where the scan position was defined at the point of best image contrast. The experimental magnification of the 12 µm radius SLG microsphere was ×3.61, and the axial position of the virtual image was −31.7 μm.

Figure 4a–d shows the captured virtual images of standard grating patterns, consisting of a 0.35-μm line width and 0.7-μm pitch at four different virtual image planes. As the scan length increased, the space between the grating patterns increased. This implies that magnifications in the image stack gradually increase according to the scan length. Figure 4e depicts the projected *x*–*z* image from the center of the image stack where the virtual image was formed. In this projection image, the edge sharpness in a specific region of interest was analyzed to obtain ${Z}_{{{\rm{v}}}_{\exp }}$. The magnification and sharpness of the grating line in each image were also analyzed, as shown in Fig. 4f. The ${Z}_{{{\rm{v}}}_{\exp }}$ of the 12-μm radius SLG microsphere was −31.7 μm, and ${M}_{\exp }$ was ×3.61. Since the obtained ${Z}_{{\rm{v}}}$ and $M$ from Eqs. (1) and (2) were −31.66 μm and ×3.60, respectively, utilizing the best image contrast was considered adequate to calculate ${Z}_{{{\rm{v}}}_{\exp }}$ and ${M}_{\exp }$. From these results, the proposed theoretical framework agrees well with the experiments. The slight differences between the experiments and the results from the geometric model were caused by using the image processing software to count pixels, where the pixels had to be converted to micrometers to obtain the total magnification of the MASR system.

**Fig. 5:**
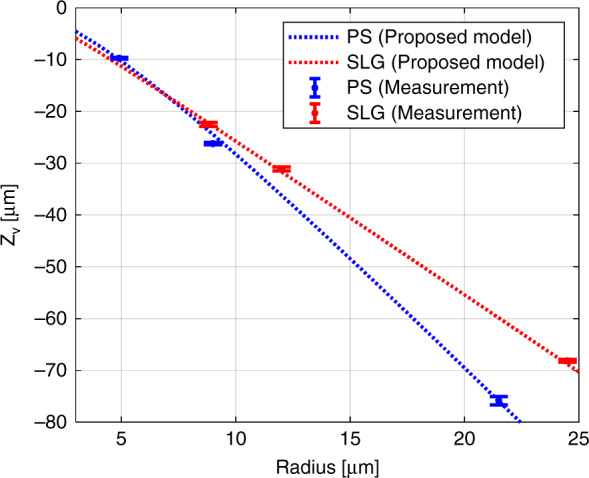
${Z}_{{{\rm{v}}}_{\exp }}$ obtained from the actual measurement and ${Z}_{{\rm{v}}}$ obtained from the proposed geometric model. Note that, ${Z}_{{{\rm{v}}}_{\exp }}$ and ${Z}_{{\rm{v}}}$ represent the relative positions of the virtual image from the sample. Two different materials were evaluated. The red-dashed line and error bars represent the SLG (*n* = 1.52 and *λ* = 524 nm) microspheres with radii of 8.8, 12.0, and 24.5 μm, and the blue dashed line and error bars represent the PS (*n* = 1.60 and *λ* = 524 nm) microspheres with radii of 4.9, 9.0, and 24.5 μm. The evaluated microspheres were commercial products from Cospheric LLC.

Figure 5 presents both the experimental and theoretical results for two different types of microspheres to verify ${Z}_{{\rm{v}}}$ in Eq. (2) when ${{\rm{w}}}_{{\rm{d}}}=0\,\upmu {\rm{m}}$. Commercially available SLG and PS microspheres with three different radii were used. ${Z}_{{{\rm{v}}}_{\exp }}$ was obtained using the same method as described in Fig. 4. In Fig. 5, the dashed line represents ${Z}_{{\rm{v}}}$ calculated by Eqs. (1) and (2), where the error bars indicate the standard deviation of ten measurements and the center value of the error bar represents the average value of ${Z}_{{{\rm{v}}}_{\exp }}$. The experimental results were found to be consistent with the results obtained from the geometric model.

In Fig. 5, the trend between ${Z}_{{\rm{v}}}$ and radius for the SLG and PS microspheres are different. Since the refractive indices of the SLG and PS microspheres were 1.52 and 1.6, respectively, the ${D}_{{\rm{f}}}$ values consequently differed even with the same radius. At the same radius, a virtual image occurred at a lower position from the PS microsphere than from the SLG microsphere, owing to the higher refractive index of PS. Therefore, the magnification of the virtual image by the PS microsphere was higher than that by the SLG microsphere.

In a previous study^33^, the best resolution was obtained with a 6-μm diameter microsphere, which generated a photonic nanojet with a minimum waist. Similar to the previous study, when the radius of the microsphere was smaller, a better resolution was acquired in the experiment. However, the FOV of the virtual image varies according to the radius of the microsphere. The smaller the microsphere radius, the smaller the FOV. From a productivity perspective, this smaller FOV means that more measurements must to be taken when using a smaller microsphere to cover a given area of the sample to be imaged, resulting in a lower throughput. In other words, there exists a trade-off between imaging resolution and FOV, similar to that in conventional optical imaging systems.

Consequently, the appropriate radius and material for a microsphere can be determined using the presented geometric model to obtain a super-resolution image with the desired FOV and magnification. Moreover, this model can provide optimal positions of optical components for non-contact measurement, which enables the application of this technique to semiconductor inspection and metrology.

### Ultra-small spot spectral measurements

The ability of the MASR system to magnify images is clear from the previous sections. Small spot spectral measurements using super-resolution were experimentally verified, as reported in this section. The measurement area and SNR were evaluated under super-resolution conditions. The influence of the microsphere on spectral reflectance is also introduced in this section and evaluated by the standard thickness sample.

The MASR system requires a reference spectrum to calculate spectral reflectance. The spectral reflectance, $R$, can be obtained by the following equations:

3 $${\left(\frac{{E}_{{\rm{out}}}}{{E}_{{\rm{in}}}}\right)}^{2}={R}_{{\rm{meas}}}=\frac{{R}_{{\rm{meas}}}}{{R}_{{\rm{ref}}}}\cdot {R}_{{\rm{ref}}}=\frac{\frac{{I}_{{{\rm{out}}}_{{\rm{meas}}}}}{{I}_{{\rm{in}}}}}{\frac{{I}_{{{\rm{out}}}_{{\rm{ref}}}}}{{I}_{{\rm{in}}}}}\cdot {R}_{{\rm{ref}}}=\frac{{I}_{{{\rm{out}}}_{{\rm{meas}}}}}{{I}_{{{\rm{out}}}_{{\rm{ref}}}}}\cdot {R}_{{\rm{ref}}}$$

where *E* denotes the electric field, *I* denotes the optical intensity, and the subscripts “meas” and “ref” refer to the target sample and reference material, respectively, the latter of which has a well-known spectral reflectance.

${I}_{{\rm{out}}}$ can be measured using the spectrometer; however, it is not obvious how to obtain ${I}_{{\rm{in}}}$. Equation (3) shows that it is possible to alternatively calculate ${R}_{{\rm{meas}}}$ by measuring ${I}_{{{\rm{out}}}_{{\rm{ref}}}}$, which is the spectroscopic intensity of the reference sample, although ${I}_{{\rm{in}}}$ still remains unknown. The reference material is necessary to calculate ${R}_{{\rm{meas}}}$ without ${I}_{{\rm{in}}}$.

#### Spot size verification

A 23-μm SLG microsphere was used to evaluate the performance of the MASR system. A ×100 objective lens with 0.9 numerical aperture (N.A.). was used to measure spectral reflectance.

**Fig. 6:**
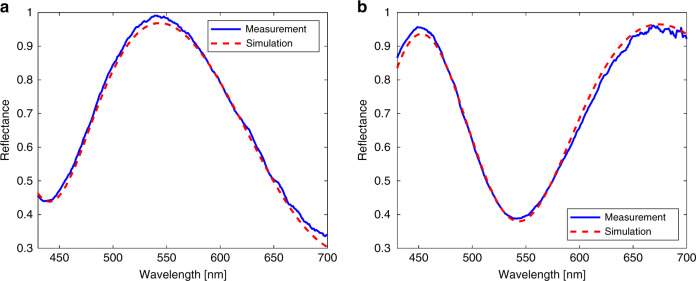
Experimental results measured by the MASR system. **a** Results for the SiO_2_ Film #1 487.1 nm obtained by the MASR system with a root mean square error (RMSE) = 0.0015 (Reference: 494.4 nm). **b** Results for the SiO_2_ Film #2 391.3 nm obtained by the MASR system with an RMSE = 0.0022 (Reference: 392.1 nm).

To verify the reflectance measurements made by the MASR system, two SiO_2_ standard film wafers were prepared and evaluated by an RC-2 ellipsometer (Woollam). Figure 6a, b depicts the results measured by the MASR system. The blue lines represent the reflectance measured by the MASR system, and the red dotted lines represent the best-fit regression result of the simulation curve calculated by the Fresnel reflectance. These results indicate that it is possible to measure the spectral reflectance, although the incident light passes through the microsphere, which has its own refractive index and volume.

**Fig. 7:**
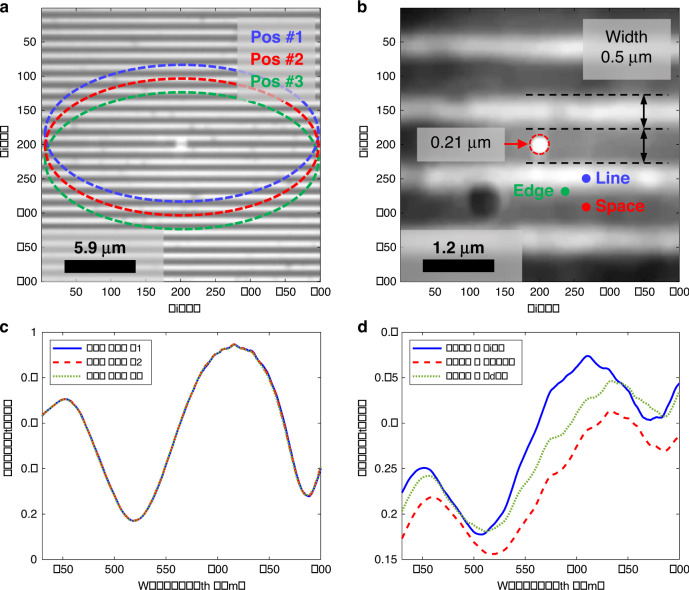
Spectral measurements for an extremely small spot obtained by the MASR system. 0.5-μm line width and 1-μm pitch grating pattern and the reflectance measurement areas (schematically superimposed) for **a** the spectroscopic ellipsometer in the ×100 image, which had a spot of major axis 25 μm for three different positions, and **b** the approximately ×530 super-resolution image obtained by the microsphere and relevant positions (line, space, and edge). **c** Spectral reflectance of the ellipsometer for the positions in **a**. **d** Spectral reflectance of the MASR system for the positions in **b**. (OCD optical CD, Pos position).

One of the great advantages of the MASR system is that the reflectance can be obtained by the super-resolution of the microsphere. Figure 7a, b depict a 0.5-μm width and 1-μm pitch grating pattern obtained by the MASR system. Spectral reflectance or optical CD was measured using an ellipsometer. Ellipsometry is generally performed with a 60–70° angle of incidence (AOI) and 25–30 μm major axis length of beam spot. In this experiment, the AOI was fixed at 60°, and the major axis length of the measurement spot was 25 μm. Measurement spots at different positions are schematically superimposed on the ×100 image in Fig. 7a because they could not be visualized in the actual measurement. The area of the spectral measurement spot can be verified using the spotlight connected by an optical fiber. By comparing the line width, spatial patterns, and spot diameter in the captured image, the pixel resolution of the MASR system and the diameter of the measurement spot can be calculated. By using the super-resolution of the microsphere, the pixel resolution was enhanced by a factor of up to ×5.3 and the spot diameter was 0.21 μm with a ×100 magnification objective and an optical fiber with a diameter of 100 μm. In other words, the measurement area became extremely small owing to approximately ×530 imaging magnification.

The spectral reflectance values of the grating patterns were measured to verify the measurement area in Fig. 7. The results indicated that the diameter of the measurement spot was smaller than the line and spatial width at 500 nm, and the spectral signal varied according to the location of the measurement spot. The result is depicted in Fig. 7d, where the spectrum varies according to the location of the grating. In the conventional ellipsometer, spectral signals do not vary with the location of the measurement spot, as the spot of the spectral measurement is larger than the width of the grating. Average values of the width and thickness can be measured by rigorous coupled-wave analysis, because the conventional ellipsometer acquires averaged signals of lines and spaces. However, it cannot determine the thickness of each grating pattern or edge area of the grating.

#### SNR enhancement

Another advantage of spectral measurements using the MASR system is SNR enhancement. The SNR of an image or a spectral signal from a detector is relatively high with high optical power at the same acquisition time because the detector has a constant level of dark noise. Commercially available objective lenses have different back focal aperture designs, which cause the beam size at the back focal plane to decrease to compensate for the side-effect of high N.A., such as aberrations. Therefore, the optical power detected by a camera or spectrum detector typically decreases at high magnifications. In other words, it is difficult to avoid SNR loss at high magnifications. However, in the case of the MASR system, the SNR loss was minimized by the photonic nanojet effect, which concentrated the incident and reflected light.

**Fig. 8:**
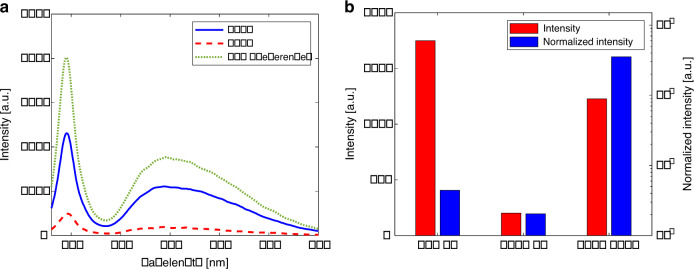
Comparison of intensity and normalized intensity for ×50 SR, ×100SR, and ×530 MASR. **a** Intensities in the wavelength range of 430–700 nm for ×50 SR, ×100 SR, and ×530 MASR. **b** Intensities and normalized intensities for ×50 SR, ×100 SR, and ×530 MASR.

Average intensities of the reflected light in the wavelength range of 430–700 nm, corresponding to each magnification, are shown in Table 1 and Fig. 8. The spectrometer counted the number of photons of each wavelength with an integration time of 100 ms. The intensity is given in arbitrary units (a.u.), which represent the optical power according to wavelength. As presented in Table 1, MASR achieved the highest pixel resolution (0.012 μm/pixel). The relative intensity to ×50 SR was also calculated. The relative intensity of MASR was 70.2%, which was significantly higher than that of the ×100 SR (10.5%) despite the ×5.3 higher magnification.

**Table 1:** Comparison of magnification, pixel resolution, spot diameter, intensity, relative intensity, and normalized intensity of the experimental results.

	×50 SR	×100 SR	MASR
Magnification	×50	×100	×530
Spot diameter (μm)	2.24	1.12	0.21
Pixel resolution (μm/pixel)	0.119	0.059	0.012
Intensity (a.u.)	1749	201	1227
Relative intensity to ×50 SR	100%	11.5%	70.2%
Normalized intensity (a.u.)	444	204	35,426

A normalized intensity was introduced to account for the different measurement area sizes under different magnifications. It was calculated as the average intensity divided by the measurement spot area, representing the signal efficiency per unit area for a given magnification. The MASR system exhibited the maximum normalized intensity and highest magnification in comparison with the other systems under the same conditions; the normalized intensity of MASR was approximately 80 times higher than that of ×50 SR. These results experimentally verify the concentration of light by the microsphere.

### Semiconductor applications

This section describes the evaluation of the semiconductor devices by the MASR system. Both super-resolution imaging and small spot spectral measurements were applied to different devices.

**Fig. 9:**
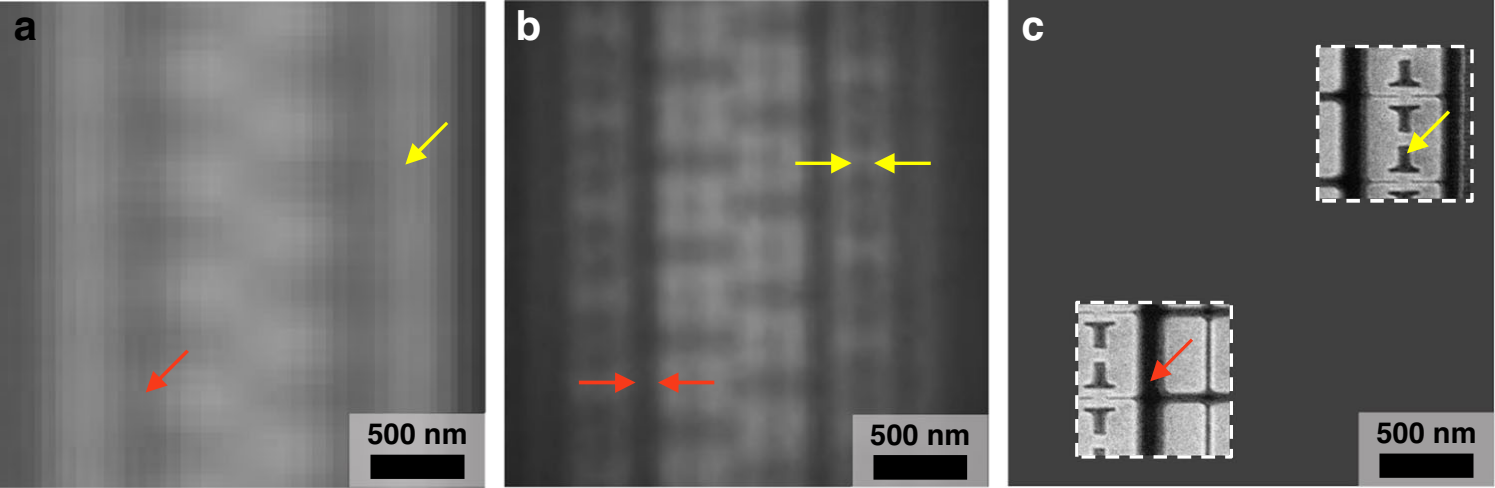
Images of the SWD area in the DRAM. Images of the SWD area in the DRAM imaged by **a** ×100 with 0.9 N.A. and **b** MASR. **c** Corresponding SEM image. 57 nm CD indicated by yellow arrows and 146 nm CD indicated by red arrows.

First, the sub-word line driver (SWD) area in a DRAM was imaged by the MASR system. This is a narrow area in the device and consists of small structures having CDs under 200 nm, which is smaller than the optical limit for conventional white-light microscopy. The Rayleigh criterion was calculated to provide an optical resolution of approximately 280 nm for a broadband light source. The 57 nm lines were distinguished by the MASR system, which were originally unresolved at ×100 magnification without the microsphere, as depicted in Fig. 9a, b. The 146 nm lines were also blurred at ×100 magnification but were clearly resolved in the MASR system. For reference, an SEM image is illustrated in Fig. 9c. The SWD area is important to control features, such as gate oxide (GOx) thickness and dent (slightly etched area) depth, which can affect the dielectric characteristics. This area is considered as a weak point in the measurement process, as it is difficult to measure this area directly with a conventional ellipsometer owing to its large measurement spot, which has a major axis length of 25–30 μm. The MASR system allows this area to be monitored with super-resolution and can locate spectral measurement spots below a 100-nm resolution.

**Fig. 10:**
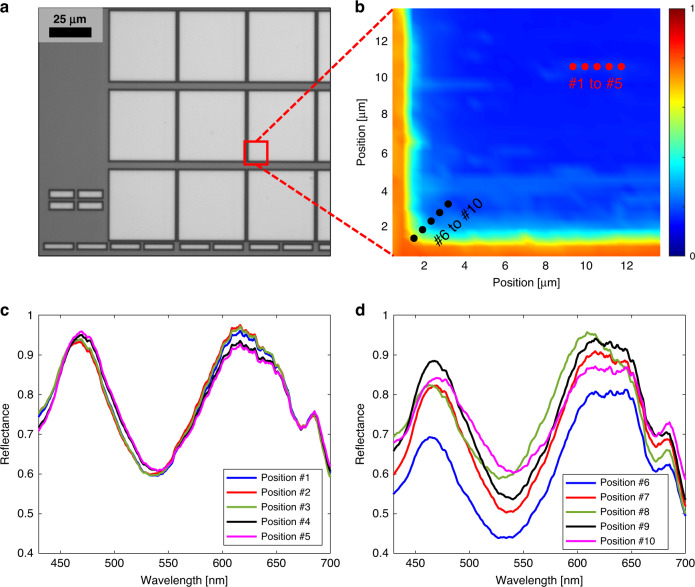
Spectrum measurement result of the edges and corner areas of cell blocks. **a** ×20 image of DRAM cell array. **b** Principal component analysis map of the reflectance obtained by MASR. The red dots (#1 to #5) refer to the center positions of the cell block, while the black dots (#6 to #10) refer to the edge area from the outside of the cell block. **c** Spectral reflectance for positions #1 to #5 and **d** #6 to #10 in **b**.

The spectral reflectance of a cell block in the DRAM depicted in Fig. 10a was evaluated by the MASR system. There has been an increasing demand for measuring the edges and corner areas of cell blocks; however, it is difficult to measure these areas using conventional spectrum systems. It is crucial to control the in-cell locality including the edges of the cell block because defects often occur at the edges during the multiple etching steps in DRAM. Sampling and destructive methods are the only ways to analyze the device after defects occur. MASR can measure the spectral reflectance in the edge area, whereas both ellipsometers and imaging spectrum systems cannot.

As depicted in Fig. 10a, b, the reflectance at the center and edge of the cell block was compared for five positions by MASR. The distance between each position was 0.5 μm. This measurement density was considered appropriate for observing the reflectance changes in the edge area, as it is known that DRAM edge defects often occur within 2 μm from the edge. The central spectral reflectance shown in Fig. 10c varied slightly between positions, indicating that there were small structural dimensional changes. Conversely, the reflectance at the edge shown in Fig. 10d significantly varied between positions, indicating substantial changes in the structure and the occurrence of either defects or imperfect structures.

The spectral map rapidly collapsed near the edge area of the cell block in Fig. 10b. This indicates the feasibility of the MASR system to monitor the edge, particularly 2 μm from the end of the cell, which cannot be measured using conventional spectrum systems. Microsphere super-resolution has the potential to be used in the semiconductor industry, which requires the measurement of a large number of steps and structures.

## Discussion

The performance of the MASR system, which can obtain an image resolution better than the Rayleigh limit with white-light illumination, was demonstrated. By utilizing the super-resolution image enhanced by a microsphere lens, it was possible to use nanospot spectral reflectance to inspect 3D structural defects. This is the first reported approach that combines spectral measurements and microsphere super-resolution imaging.

A super-resolution capability of approximately ×2 was verified by resolving a 57-nm feature, while a conventional microscope could not resolve patterns smaller than 274 nm in the visible wavelength range of 430–700 nm. The MASR system could achieve a total magnification of approximately 530X, resulting in an image sensor resolution of 0.012 μm/pixel. A geometric model was introduced to explain the magnification rule for the microsphere super-resolution, by combining an FDTD simulation and the theory of classical ray optics. This model could aid in selecting the appropriate microsphere and optimal contactless positions of the objective lens and microsphere to obtain a high-contrast image with the desired magnification. It was possible to monitor features under 100 nm in the SWD area of DRAM by using MASR, which could not be resolved using conventional white-light microscopy. Structures under 100 nm could be resolved without an immersion medium and without contact.

In addition to the super-resolution imaging, the spot of the spectral measurement was successfully reduced to a diameter of 210 nm using the MASR system. To date, most studies have focused on super-resolution capabilities; however, the MASR system extends the usefulness of the photonic nanojet effect to spectroscopy metrology. The measurement spot is ×119 smaller than that of conventional spectral systems, which have major axes with a length of 25–30 μm. Furthermore, the MASR system achieved a spectral resolution of under 1 nm in the visible wavelength range. Conventional imaging spectrum systems, which require a change in the wavelength of the incident light, are limited by their low spectral resolution. There have been increasing demands for direct spectral measurements to investigate nanostructure changes and the locality of semiconductor devices because test element group patterns cannot represent cell patterns. However, conventional spectrum systems cannot satisfy these demands owing to their lower spectral resolution or larger measurement spot. The MASR system demonstrated great advantages over conventional systems owing to its small measurement area. Spectral changes at the edge of the cell block were successfully monitored using the MASR system. Furthermore, the SNR losses were suppressed despite the extremely high magnification.

Nevertheless, several points should be further considered for use in in-fab metrology equipment. Precise control of short working distances (under 1 μm) is necessary. The microsphere must be located at a proper position and the distance between the microsphere, objective, and sample needs to be robustly maintained. Additionally, sensitivity enhancement needs to be studied.

The proposed technique can be applied to conduct non-destructive, direct measurements of various semiconductor devices including Logic SRAM and local areas of DRAM. Furthermore, MASR can be easily applied to various optical measurement systems. It can enhance the optical resolution by ×2 and add an additional ×4 to ×5 magnification to any white-light-based imaging system at a low cost. To the best of our knowledge, this is the world’s first demonstration of a novel system concept to overcome the current metrology challenges.

## Material and methods

### FDTD simulation and ${D}_{{\rm{f}}}$

The location of the photonic nanojet generated by microspheres with different diameters and materials was obtained using the MEEP toolkit, which can also be used to perform FDTD simulations. A plane wave with a wavelength of 547 nm, the central wavelength of the white-light LED used in the hands-on system, propagated downward to the microsphere, forming a photonic nanojet on the far side of the microsphere. Various microspheres, including those with radii of 2.5, 5, 10, and 20 μm, for SLG (*n* = 1.52) and PS (*n* = 1.6) were simulated using MEEP to determine the distance between the microsphere and photonic nanojet, ${D}_{f}$. *n* represents the refractive index for a wavelength of 532 nm. ${D}_{f}$ for each radius was calculated by linear interpolation including a constant term for calibration depending on the refractive index.

### Vertical scanning and calculation of sharpness score and magnification

Vertical scanning images could be obtained using the MASR system by employing the PZT scanner comprising a PZT actuator and crafted flexure-hinge system. The PZT actuator (custom product, Physik Instrumente, Germany) had a travel range of 150 μm, with a resolution of 1 nm and linearity error of 0.01% in the travel range. The push/pull full force capacities of the actuator were 3000 and 700 N, respectively. The mass of the lens turret was approximately 1 kg including the objective lenses. The scanning system included a flexure-hinge connected with the actuator (SNU Precision, South Korea), which could move the lens turret with multiple objective lenses precisely and stably. The system improved the push/pull forces of the PZT actuator, enabling the system to move the lens turret vertically with sufficient force for stable high-speed scans.

The measurement scan range was 80 μm and the scan interval was 0.08 μm in the section “Semiconductor applications.” Consequently, the total number of images in one image stack was 1000, and this range could cover the range of vertical positions from the original image by the objective lens only to the magnified virtual image. By using a projected x-z image in the image stack, the background signal was removed by calculating the median value of the moving kernel. The position of optimal focus was determined by calculating the edge sharpness using the Sobel filter. The Sobel filter uses two 3 × 3-pixel kernels that are convolved with the original image in the *x*- and *y*-directions. At each point in the image, the sharpness score was defined as the gradient magnitude between two kernels and was calculated at the center of the virtual image in each *x*–y image of the 3D image stack. The optimal distance of the virtual image plane where the averaged sharpness score was maximized in the *z*-direction was defined as the vertical position of the best focus.

The total magnification ${M}_{{\rm{total}}}$ described in the section “Experimental results” can be expressed as follows:

4 $$\begin{array}{ll}{M}_{{\rm{total}}}={M}_{{\rm{optics}}}\times {M}_{\exp }=\frac{{\rm{image}}\,{\rm{width}}\,}{{\rm{object}}\,{\rm{width}}}\\ \qquad\quad=\frac{{\rm{number}}\,{\rm{of}}\,{\rm{pixels}}\,{\rm{in}}\,{\rm{image}}\times {\rm{pixel}}\,{\rm{size}}\,}{{\rm{object}}\,{\rm{width}}}\end{array}$$

where ${M}_{{\rm{optics}}}$ is the optical system magnification, ${M}_{\exp }$ is the measured microsphere magnification, and “image” in Eq. (4) means the super-resolution image having magnification ${M}_{{\rm{total}}}$. Therefore, ${M}_{\exp }$ in the super-resolution image acquired by the MASR system can be obtained by

5 $${M}_{\exp }=\frac{{\rm{number}}\,{\rm{of}}\,{\rm{pixels}}\,{\rm{in}}\,{\rm{image}}\times {\rm{pixel}}\,{\rm{size}}}{{\rm{object}}\,{\rm{width}}\times {M}_{{\rm{optics}}}}$$

In this study, the object was a standard grating pattern of 0.35 μm line width and 0.7 μm pitch. The pixel size is defined as the width of a single charge-coupled device pixel. The pixel size of the camera was 6.5 µm (Panda 4.2, PCO, Germany). A ×20 objective lens (LMPLFLN, Olympus, Japan) and ×1 tube lens (custom product, SNU Precision, Korea) were used. The data were processed and analyzed with Matlab (MATLAB R 2019A, MathWorks, Inc., USA) and ImageJ software (provided in the public domain by the National Institutes of Health, USA; http://imagej.nih.gov/ij/).

For MASR imaging, commercial microsphere products were used from Cospheric LLC, USA (PSMS-1.07 9.5–11.5 μm, PSMS-1.07 14–20 μm, PSMS-1.07 38–48 μm, S-SLGMS-2.5 15–19 μm, S-SLGMS-2.5 23–26 μm, S-SLGMS-2.5 48–51 μm). The exact radii of the microspheres had to be measured, as the product was provided only with a range of radii. This could be measured by the MASR system, which could also calculate the lateral dimensions of the specimen. For MASR imaging, the microsphere was attached to a 10 µm tip of a glass micropipette (Fivephoton Biochemicals, USA) using a UV-curable optical glue (NOA81, Thorlabs, USA). The pipette was mounted in a micromanipulator (the Patchstar from Scientifica, United Kingdom), which could translate in the *x*-, *y*-, and *z*-axes.
